# A Review of the Literature: Amniotic Fluid “Sludge”—Clinical Significance and Perinatal Outcomes

**DOI:** 10.3390/jcm13175306

**Published:** 2024-09-07

**Authors:** Sonia-Teodora Luca, Vlăduț Săsăran, Mihai Muntean, Claudiu Mărginean

**Affiliations:** 1 Department of Obstetrics and Gynecology, Clinical County Hospital of Mureș, Samuel Köteles Street No. 29, 540057 Târgu Mureș, Romania; sonialuca11@gmail.com (S.-T.L.); munteanmihai@yahoo.com (M.M.); marginean.claudiu@gmail.com (C.M.); 2 Department of Obstetrics and Gynecology, Faculty of Medicine in English, George Emil Palade University of Medicine, Pharmacy, Sciences, and Technology of Târgu Mureș, Gheorghe Marinescu Street No. 38, 540136 Târgu Mureș, Romania; 3 Department of Obstetrics and Gynecology, Faculty of Medicine, George Emil Palade University of Medicine, Pharmacy, Sciences, and Technology of Târgu Mureș, Gheorghe Marinescu Street No. 38, 540136 Târgu Mureș, Romania

**Keywords:** sludge, amniotic fluid, preterm birth, chorioamnionitis, cervical insufficiency

## Abstract

**Introduction:** This paper seeks to report and emphasize the most important aspects from the scientific literature about the diagnostic accuracy of the amniotic fluid “sludge” (AFS), its characterization, its treatment, and its association with premature birth. AFS is defined as a floating freely hyperechogenic material within the amniotic cavity in the proximity of the internal os. **Materials and Methods:** We conducted a search on Pubmed and Google Scholar for relevant articles on the subject of amniotic fluid “sludge” published until January 2024. Searches were focused on articles about diagnosis, treatment, maternal and neonatal outcomes, risk of preterm birth, and case reports. The full-text reading stage resulted in the inclusion of 51 studies. **Results:** AFS is independently associated with chorioamnionitis, preterm delivery, short cervix, increased risk of neonatal morbidity, and cervical insufficiency. This hyperechogenic free-floating material is linked with preterm birth before 32 weeks of gestation, especially when it is associated with short cervical length. **Discussion:** Present studies identify some controversial benefits of antibiotics in reducing the incidence of preterm birth in women with AFS. Nevertheless, in this review, we can conclude that the presence of AFS in pregnancy is a marker for the microbial invasion of the amniotic cavity, as it is associated with preterm birth. Further studies on a larger group of patients are necessary to clarify and exactly define the terms of managing these cases.

## 1. Introduction

Nowadays, preterm birth or childbirth before 37 weeks of gestation is one of the leading causes of neonatal mortality and morbidity [1]. The etiology of preterm birth is multifactorial and intensive research efforts are being made in order to better understand the underlying causes [2].

**Figure 1:**
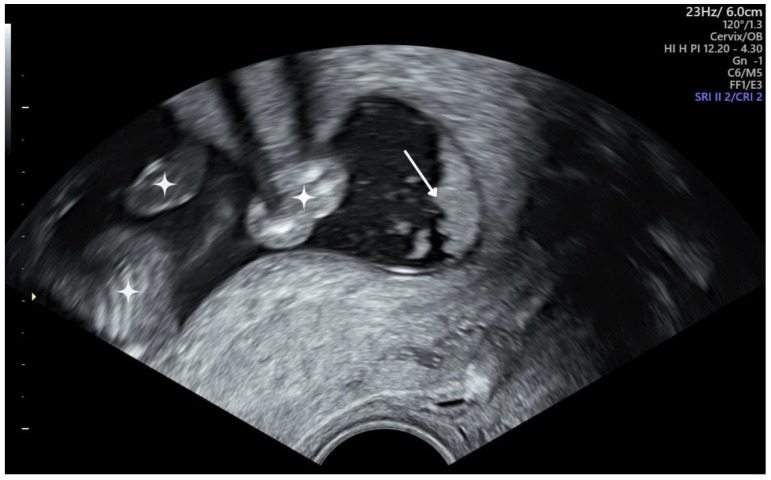
Amniotic fluid “sludge” in a 17—week-pregnant woman during transvaginal ultrasound. The image is illustrative for funneling in the cervical os. Legend ⟡—fetal parts; →—points towards amniotic fluid sludge. Image source: personal library.

One of its risk factors is the ultrasonographic presence of amniotic fluid “sludge” (AFS), which has been reported in various studies as an independent risk factor for preterm delivery occurring before 28, 32, and 35 weeks of gestation and also before 34 weeks of gestation in high-risk patients [3,4,5,6]. Amniotic fluid “sludge” can be found in women with intact membranes and is defined as a floating freely hyperechogenic, dense, homogenous material within the amniotic cavity in the proximity of the internal os, and is represented in Figure 1 [4,7,8].

Its presence can be observed through transvaginal ultrasonography [9,10]. Actually, AFS is present in almost 4% of pregnancies during transvaginal ultrasound in the first and second trimester. The prevalence increases with rising gestational age, reaching 88% by 35 weeks, according to Kusanovic’s study [8,11].

This “sludge” is usually caused by bacterial or fungal intra-amniotic infection. Microorganisms, such as Mycoplasma hominis, Ureaplasma urealyticum, Candida albicans, and Streptococcus mutans are common in intra-amniotic infections [8,10,12]. Their invasion in the amniotic fluid can be retrieved in women with cervical insufficiency, preterm birth with intact membranes, premature rupture of membranes, chorioamnionitis, spontaneous labor at term with intact membranes, and idiopathic vaginal bleeding [13,14,15].

Clinically, the presence of amniotic fluid “sludge” is independently associated with chorioamnionitis, preterm delivery, short cervix, increased risk of neonatal morbidity, and cervical insufficiency [7].

A systematic review conducted by Pergialiotis et al., which focused on the comparison of pregnancy outcomes among women with and without AFS, showed higher rates of deliveries before 37 weeks of gestation in patients with sludge in the amniotic fluid, lower gestational age at delivery, and lower neonatal birthweight with an increased risk of neonatal death. The review also highlighted the limitations of evidence about neonatal morbidity in present studies. Moreover, the clinical implications of AFS remain unknown and the direction of causation could not be determined because the majority of studies included in their systematic review did not investigate it [16]. In 2023, another review by Pannain et al. underlined the limitations of the studies published before and suggested that more evidence is needed regarding the treatment of AFS and its association with prematurity [17]. Previous reviews on this subject have solely focused on the diagnosis or treatment of AFS and highlighted the population characteristics of the available studies without including all the available studies analyzing the role of AFS in premature birth at the time of publication. Yet, studies focusing on aiding in a better identification of amniotic fluid “sludge”, and risk factors for its development and treatment, are still scarce.

This paper seeks to report and emphasize the possible association between ultrasonographic features of AFS and premature birth. Secondarily, we aim to highlight the diagnostic accuracy and characterization of AFS and its management.

## 2. Materials and Methods

We conducted a search on Pubmed and Google Scholar for relevant articles on the subject of amniotic fluid “sludge” published until January 2024. Terms used to sort relevant data were “amniotic fluid AND sludge”, “preterm AND birth”, “intraamniotic AND infection”, “amniotic sludge”, “echogenic AND particles”, “intraamniotic AND sludge”, and “treatment AND amniotic AND fluid AND sludge”. The abstracts of all the papers found in the previously mentioned databases were analyzed; those that were relevant for the chosen topic were selected, and their full text was reviewed. Only population-based studies, randomized controlled trials (RCTs), and case reports were included in our review. Papers that only mentioned the above detailed search terms without focusing on this subject were excluded, as well as in vitro or animal-based studies, reviews, meta-analyses, letters to editors, commentaries, and papers that did not have an available abstract or were written in a language other than English. Moreover, the bibliography of each paper was revised in order to identify other potentially relevant sources. The study population presented in the selected articles included women with singleton pregnancies or twin pregnancy out of labor with the ultrasound diagnosis of intra-amniotic “sludge” confirmed. The following information was retrieved from the articles which made the following selection pool: data focusing on diagnosis concerns and establishment, treatment, maternal and neonatal outcomes, risk of preterm birth, and association of AFS with cervical insufficiency.

## 3. Amniotic Fluid “Sludge”: Causes and Its Association with Intra-Amniotic Infection

The full-text reading stage resulted in the inclusion of 51 studies, each of them detailed in this paper. In pregnancy, the inflammatory and bacterial infection processes lead to the production and release of proinflammatory cytokines such as tumoral necrosis factor alpha (TNF-α) and interleukins (ILs) (IL-1,IL-2,IL-3,IL-4,IL-6,IL-7,IL-8,IL-10). These cytokines stimulate and induce the production of proteases and uterotonins (prostaglandins and metalloproteinases), which can cause uterine contractions and can weaken the membranes and determine their premature rupture. Therefore, this mechanism is thought to be associated with the presence of amniotic “sludge” too [7,17,18,19,20].

AFS is also known as a material that reflects the presence of biofilms inside the amniotic fluid, and in 2008, Romero R. et al. identified specific bacteria inside the ultrasound visible “sludge”, which significantly aided diagnosis and therapy. In order to prove this, amniotic material was aspirated by transvaginal needle amniotomy under ultrasound guidance in patients with chorioamnionitis and preterm birth. Afterwards, scanning electron microscopy, amniotic fluid cultures, white blood cell count quantification, the histologic examination of the placenta, and fluorescent in situ hybridization (FISH) with confocal laser scanning microscopy were performed [21].

In 2018, Yoneda et al. conducted a retrospective study which emphasized that AFS is related to intra-amniotic inflammation, but not to intra-amniotic infection. This was the first study which used a Polymerase Chain Reaction (PCR)-based method to detect bacteria in pregnant women with amniotic fluid “sludge”. Moreover, the results of the study support the concept that AFS increases the risk of preterm delivery and poor neonatal outcomes (higher percentage of admissions to the neonatal intensive care unit, higher rates of respiratory distress syndrome (RDS) and bronchopulmonary dysplasia (BPD) when “sludge” was present in the amniotic fluid) [22].

In 2022, a cross-sectional study included 25 patients with gestational ages ranging between 18 and 41 weeks and the presence of hyperechogenic material in the amniotic fluid showed that this material is frequently associated with an inflammatory process. To prove this theory, the material was transvaginally retrieved with a needle of amniotomy under direct visualization. Afterwards, cultures for aerobic/anaerobic bacteria and white blood cell count measurements were performed [10].

A cohort study of asymptomatic pregnant women with sonographic short cervix (≤25 mm) and AFS who underwent amniocentesis (*n* = 62) characterized the inflammatory response by assessing the concentrations of 33 proteins using a validated multiplex assay and comparing those who delivered ≤32 and >32 weeks. The rate of preterm delivery ≤32 weeks was 56.5%, and in these patients the rate of intra-amniotic inflammation, 31.4% vs. 3.7%; *p* = 0.008, and acute histologic chorioamnionitis, 75% vs. 32%; *p* = 0.002, was significantly higher than in those who delivered after 32 weeks. In terms of mean concentrations, differences between concentrations were found for 8 out of 33 proteins when comparing those from women who delivered before 32 with those who delivered after 32 weeks. Interleukin-8 showed the highest sensitivity and specificity [23].

Patients with AFS had a higher frequency of histological chorioamnionitis, 77.8% (14/18) vs. 19% (11/58), *p* < 0.001, and positive amniotic fluid cultures, 33.3% (6/18) vs. 2.5% (1/40), *p* = 0.003, than those without the presence of “sludge” in the amniotic fluid. The presence of “sludge” was an independent factor occurrence for positive cultures from amniotic fluid and histological chorioamnionitis [8]. Moreover, the association between AFS and histological chorioamnionitis, RR: 2.34; 95%CI: 1.7–3.12, is well known [17].

A series of four cases with cervical incompetency and bulging membranes published in 2016 showed that all patients who presented with shortened cervical length and amniotic fluid “sludge” near the cervix had histological chorioamnionitis and funisitis. The findings support the presence of AFS as a useful marker of microbial invasion of the amniotic cavity (MIAC), but the study is limited due to the small number of cases included [11].

Nevertheless, within Ventura et al.’s study there were no significant differences in the association between amniotic fluid “sludge” and histological chorioamnionitis. From their study group of 16 patients, 56% of the newborns were admitted to neonatal intensive care unit (NICU) versus 23.8% belonging to the control group (48 patients without amniotic “sludge”) [3]. Within the same study, it was found that women who had “sludge” in the amniotic fluid delivered sooner than those who had none, 30.1 ± 4.5 vs. 33.1 ± 0.5 weeks, *p* = 0.01. Furthermore, the risk of preterm birth before 28 weeks of gestation was significantly higher in women with amniotic fluid “sludge”, but no differences in preterm delivery rates were seen before 34 weeks. The premature rupture of membranes was found in 50% (8/16) of the women with sludge versus 16.7% (7/42) women without “sludge” [3].

Patients with “sludge” had a significantly higher frequency of amniotic fluid cultures and histological chorioamnionitis than those without “sludge”. Newborns belonging to patients with amniotic “sludge” were admitted more often to the neonatal intensive care unit and their morbidity rate was higher than the one of newborns from mothers belonging to the control group [8].

## 4. Differential Diagnosis of Amniotic Fluid “Sludge”

The differential diagnosis of AFS during ultrasound includes intra-amniotic hematoma and subchorionic hemorrhage, which are also associated with a higher risk of preterm delivery [9,24,25]. Previous studies showed that high-density intra-amniotic particles were also possibly related to meconium, vernix caseosa, and intra-amniotic bleeding [26].

There is still no agreement regarding the diagnosis of sludge at ultrasound, which is often confused with meconium and vernix caseosa, especially when the ultrasound is performed in the last trimester [26]. Considering this aspect, the use of ultrasound for the diagnosis of AFS requires more studies in order to establish some relevant criteria.

## 5. The Impact of “Sludge” on Women with Short Cervix and It’s Association with Preterm Birth

Cervical insufficiency or the cervix which is unable to remain closed during pregnancy has been defined by transvaginal ultrasound cervical length < 25 mm before 24 weeks in women with prior pregnancy losses or preterm births at 14 to 36 weeks, or by cervical changes detected on physical examination before 24 weeks of gestation [27,28,29].

Acute cervical insufficiency is frequently associated with subclinical intra-amniotic inflammation and infection [30].

Previously published reports describe an association between the presence of amniotic “sludge” and an asymptomatic short cervix (15 mm) [8,11].

A review from 2023 published in Brazil by Pannain et al. brought forward the heterogeneity of the published studies and noticed that the presence of “sludge” in the amniotic fluid was an important risk factor not only for premature labor and delivery in high-risk patients, but also for pregnant women with a short cervix [17].

To determine the frequency of AFS in women undergoing cervical length measurement and to study the relationship between AFS and cervical length, a prospective study was conducted on women who underwent this measurement from June 2007 to March 2012. A total of 1574 cervical length measurements were performed in 1220 women and 1268 pregnancies. Out of these, 90 women had “sludge”, which accounts for only 7.1%. Furthermore, 63 cases with “sludge” and 63 controls were analyzed and the results highlighted that the identification of “sludge” during cervical ultrasound is associated with an increased risk of preterm delivery [31].

When performing transvaginal ultrasounds, some patients can present shorter cervical length along with AFS. Cervical length under 15 mm was present in 58.3% (49/84) of the patients in the study of Kusanovic et al. According to this study, patients with “sludge” had a significantly lower gestation age at delivery and lower birth weight compared to those without sludge. The presence of AFS had no correlation with the white blood cell count [8].

Boyer et al. sought to determine the clinical significance of amniotic fluid “sludge” in twin pregnancies with a short cervix. The study included 78 twin pregnancies. The prevalence of “sludge” was reported in 34.6% (27 of 78) of women. The presence of AFS in twin pregnancies with a short cervix was supported as a risk factor for extreme prematurity. Both twins had higher rates of histological chorioamnionitis in the presence of AFS, but only twin A had higher rates of funisitis and neonatal morbidity [32]. Regarding twin pregnancies, Spiegelman et al. also conducted a retrospective cohort study to evaluate the independent association of a short cervical length and amniotic fluid “sludge” with spontaneous preterm birth and concluded that a short cervical length with positive fetal fibronectin and “sludge” at ultrasound are independently associated with the risk of spontaneous preterm birth [33].

In order to determine the association between short cervix, preterm birth, and the sonographic presence of amniotic fluid “sludge”, 149 pregnant patients hospitalized in The Philippines between 18 and 29 gestational weeks were investigated. Of these, 13 only presented a short cervix and 17 were only diagnosed with amniotic fluid “sludge”, while 86 of them presented both conditions. Therefore, this study showed that the risk for a patient with a short cervix to have preterm labor was two times higher than in counterparts with normal cervix length, OR = 2.62; 95% CI = 1.23 to 5.61; *p* = 0.006. Among patients with AFS, the risk for preterm birth was also two times higher than in those without AFS. When a short cervix was associated with AFS, the risk for preterm birth increased three times [34]. To identify and characterize the risk factors for the development of AFS in women undergoing exam- or ultrasound-indicated cerclage, a case-control study was conducted. The presence or absence of “sludge” was confirmed through review of ultrasonographic images. A total of 111 women were included, 33 (29.7%) of them having sonographic evidence of “sludge”. There was no statistically significant difference in terms of maternal race, ethnicity, parity, drug/alcohol/cigarette use, body mass index, history of preterm birth, cervical length, and cervical dilation prior to cerclage placement in patients with AFS and those without. In this study, Syeda et al. underlined that the presence of “sludge” does not predict adverse neonatal outcomes and there were no significant risk factors associated with development of “sludge” among patients with exam- or ultrasound-indicated cerclage [35].

In 2023, Stuff at al. conducted a retrospective cohort study on pregnant women with a short cervical length, under 25 mm, focusing on the effect of AFS presence. The results revealed that women with AFS were more likely to have a short cervix and their risk of delivery before 24 weeks of gestation was higher. Furthermore, in pregnant women with AFS detected during transvaginal ultrasound, an increased level of fetal fibronectin and cervicovaginal interleukin 8 was found, which highlighted the concept of AFS as a marker of inflammatory response [20].

The results of a study conducted in 2018 support the association between AFS and a higher frequency of a short cervix. This was the first study which showed that AFS is associated with intra-amniotic inflammation, but not with infection. All patients were evaluated using a highly sensitive nested Polymerase Chain Reaction (PCR) method for microorganisms. However, in the group of women with identifiable AFS, no significant differences were observed in cervical lengths between PCR-positive and -negative cases. These results suggest that AFS is an independent risk factor for a short cervix [22].

It has been demonstrated that AFS has a direct effect on the cervical length of women with pregnancies obtained after IVF procedures [36]. Moreover, the same paper which presented this novel correlation highlighted the fact that preterm delivery is probable in the presence of AFS, despite preventive treatment such as cerclage [37].

A retrospective cohort study was conducted among singleton pregnancies after cervical cerclage by Y. Huang et al. in 2022. Its purpose was to evaluate the relationship between amniotic fluid “sludge” and/or short cervical length (≤25 mm). The study revealed that AFS and a short cervix have a direct effect on pregnancies after cerclage. The association between the presence of AFS and small gestational age at delivery was statistically significant in women with a cervical length ≤ 25 mm. The proportions of patients with a cervical length ≤ 25 mm and AFS after cerclage that had preterm birth at <28, <32, and <36 weeks were higher than those without AFS. [27].

In agreement with the studies mentioned before, Himaya et al. found that the hyperechogenic free-floating material from the amniotic fluid called “sludge” is linked to spontaneous preterm birth before 32 weeks of gestation, especially when it was associated with short cervical length [38]. To determine whether intra-amniotic “sludge” correlates with preterm delivery in patients with cervical cerclage, a retrospective cohort study of patients who had undergone McDonald cerclage was conducted. In a study enrolling a total of 177 patients, sixty patients had sonographic evidence of “sludge” and there was no significant difference in the mean gestational age at delivery between the two study groups, as well as no statistical difference in the rate of preterm birth at <28, <30, <32, or <36 weeks of gestation. The authors concluded that the intra-amniotic presence of “sludge” on ultrasound is not associated with an increased risk of preterm birth in patients with cervical cerclage [39].

All the studies summarized in Table 1 highlighted an increased risk of preterm birth in pregnant women with amniotic “sludge” present at ultrasound, as well as in patients with a short cervix.

Despite all of the results obtained, in 2019, in Thailand, Kovavisarach et al. studied the prevalence of AFS in low-risk patients and concluded that prevalence of AFS in all cases was 72/330 (21.8%) and premature delivery occurred more frequently in patients without AFS. According to them, transvaginal ultrasound demonstrated the presence of AFS, but was not sensitive for screening of preterm birth [44].

To evaluate the impact of AFS on the risk of preterm delivery, in 2015, Fuchs at al. conducted a case-control study whose results showed that patients with AFS were more likely to be obese and to have had previous preterm delivery, second trimester vaginal bleeding, and cervical cerclage than those from the control group who received treatment with azithromycin, and was associated with a higher risk of premature birth. They also discovered that azithromycin treatment could reduce the risk of preterm delivery before 34 weeks. However, Fuchs et al. reported that the increased risk of preterm delivery does not correlate with the presence of “sludge” [40].

## 6. Treatment Options and Their Efficacy

Considering the association with a high risk of preterm birth and its subsequent maternal and neonatal complications, the presence of amniotic “sludge” should be treated. According to the studies which are currently available, its treatment consists of oral antibiotics. Azithromycin and Moxifloxacin were the most widely used in many studies and the results showed that the use of certain antibiotics did no treat or improve the subsequent adverse obstetric and neonatal outcomes [7]. The data analyzed in 2023 by Giles at al. did not support the use of Azithromycin in women with a short cervix, including in those with detectable AFS, because the risk of preterm birth was higher in the antibiotic-treated group [45].

In order to determine if Azithromycin, administered in cases of shortened cervix, results in the prolongation of gestation and improves neonatal outcomes, a retrospective cohort study was performed at three tertiary maternity services in Melbourne, Australia. From a total of 374 women included in the study, 129 received Azithromycin and 245 received no antibiotics. The risk of preterm birth was, overall, higher in the treatment group, 95% confidence interval 1.04–1.77, *p* =  0.023, with no differences found for the premature rupture of membranes, chorioamnionitis, or neonatal morbidity. The data collected in this study did not support the routine use of Azithromycin in women with a short cervix, including in those with AFS [45].

According to Cuff et al.’s study, antibiotic treatment was not associated with a reduction in premature birth between the treated and untreated women. Moreover, the study explains that there were no differences in the incidence of premature rupture of membranes. Regarding the resolution of amniotic fluid “sludge”, this was seen mostly in the untreated group (43%), which signifies that the antibiotic therapy did not influence the persistence of “sludge”. Moreover, mean gestational age at delivery was also not influenced by the antibiotic treatment [7].

In contradiction with Cuff’s study, Wan Wu Jin et al. conducted a retrospective study in 2021 which sought to observe the effect of antibiotic treatment on women with AFS at 15–32 weeks of pregnancy who presented uterine contractions and intact membranes. The following antibiotics were used as part of the treatment: Ceftriaxone, Clarithromycin, and Metronidazole. Moreover, all patients included in the study used tocolytics. Transvaginal ultrasound examination was performed to measure the cervical length and to evaluate the presence of AFS. Afterwards, based on the resolution or persistence of AFS, the patients were divided into two groups. Women who received treatment and in whom the disappearance of “sludge” was encountered delivered later. Therefore, they experienced a lower rate of preterm birth. The maximum duration of antibiotic administration was 4 weeks [46].

Antibiotic therapy for AFS was also studied by Hatanka et al. within an observational, controlled study which presented the benefits of antibiotics in reducing the incidence of preterm birth in women with high risk and amniotic “sludge”. Moreover, the study emphasized the impact of treatment with antibiotics on the newborns’ weight. The birth weight was significantly different between the study groups (higher for the group who received antibiotic treatment) [47].

In 2019, Dinglas et al. reported a case of a 33-year-old woman with confirmed histologic chorioamnionitis and intra-amniotic “sludge”. The results were positive for Ureaplasma parvum and bacterial vaginosis. At 19 weeks, the patient had a cerclage and received treatment with Metronidazole (5 days) and afterwards Azithromycin and Amoxicillin (14 days). Follow-up ultrasounds demonstrated the resolution of sludge, and this case provided evidence of successful treatment with antibiotics [48].

Pustotina performed a prospective study in 2020 which aimed to evaluate the association of oral and intravenous antibiotic treatment with vaginal antibiotics, vaginal progesterone, and indomethacin in pregnant women between 15 and 24 weeks of gestation with the presence of amniotic fluid sludge at transvaginal ultrasound. Vaginal Progesterone and Indomethacin were used in women with a short cervix (<25 mm). After the first and second week of therapy, all patients underwent a follow-up scan to evaluate the AFS resolution and for re-assessment of cervical length. The study asserted the beneficial effects of antibiotic treatment: the disappearance of “sludge” in all study participants. Furthermore, it underlined the histological association of sludge with chorioamnionitis and funisitis, and the hypothesis that amniotic fluid ‘sludge’ represents a marker of intra-amniotic infection [49,50].

Our review included five studies focusing on the importance of antibiotic treatment, as presented in Table 2.

While Cuff’s study did not show improvements in the risk of preterm birth and neonatal outcomes in patients treated with Azithromycin and Moxifloxacin, Hatanka et al.’s, Pustotina et al.’s, and Jin et al.’s studies pointed out the beneficial effect of antibiotic treatment. In these studies, antibiotic therapy was associated with a reduced incidence in preterm birth and neonatal morbidity. The resolution and disappearance of sludge at ultrasound was found in Pustotina et al.’s and Jin et al.’s studies after treatment. The use of Azithromycin is controversial, and current studies highlight the fact that its use does not reduce the risk of preterm delivery, nor does it produce changes for the better [7,46,47,49].

In comparison, Sapantzoglou et al. performed a meta-analysis of four retrospective cohort studies which summarized the impact of antibiotic therapy on preterm birth rates in women diagnosed with AFS. The findings suggested that the use of antibiotics does not benefit the prognosis of premature birth, and that antibiotics are associated with a statistically significant reduction in preterm labor before 28, 32, and 34 weeks [51].

## 7. Conclusions

The topic of this review is still controversial because of the reduced number of documented cases and studies. This review of the literature aimed to underline and summarize the most important aspects currently known about amniotic fluid “sludge” in order to facilitate better diagnosis and treatment. Therefore, this review based on 50 studies demonstrated a high prevalence of preterm birth among patients with AFS and highlighted that AFS is an independent risk factor for preterm birth among high-risk patients, especially in those with a short cervix.

We can conclude that the presence of intra-amniotic “sludge” in pregnancy is a marker of the microbial invasion of the amniotic cavity and is associated in the majority of cases with intra-amniotic infection. The accurate identification of sludge during the last trimester ultrasound can still be confused with artifacts mimicking its sonographic appearance. However, while still a controversial topic, ultrasonographic AFS detection appears to be a reliable marker of intra-amniotic infection.

In order to prevent premature birth and to prolong pregnancy further, studies on a larger group of patients are necessary to investigate the presence of “sludge”. Some studies presented the benefits of antibiotics in decreasing neonatal morbidity and the incidence of preterm birth in women with high risk and amniotic “sludge”, but a proper management and treatment scheme for this was not established. Taking this into consideration, it becomes clear that further evidence and studies are needed for better diagnosis and treatment.

## Figures and Tables

**Table 1:** Systematic table of neonatal outcomes and risk of preterm birth. Legend of Table 1: AFS = amniotic fluid sludge; PTB = preterm birth; vs. = versus; PROM = premature rupture of membranes; NICU = neonatal intensive care unit; CL = cervical length; IVF = in vitro fertilization.

Table 1. Reference (Author and Year)	Type of Study	Population and Study Group Assignment	Cervical Length	Outcomes -Preterm Birth- -Neonatal Outcomes-
Ventura et al. (2011) [3]	Retrospective cohort study	64 patients included		-Women from the study group delivered earlier than women from the control group -The rate of spontaneous PTB—study group vs control: within 48 h-25% vs. 2.4%; within 7 days-37.5% vs. 11.9%; within 14 days-75% vs. 23.8%; -PROM-in 50% vs. 16.7% -56% of newborns from study group were admitted to NICU vs. 23.8% from the control group
		16 with AFS—study group	≤25 mm-in 75% of patients with AFS ≤15 mm-37.5%	
		48 without AFS—control group		
Hatanaka et al. (2016) [6]	Prospective cohort study	195 patients	<25 mm-21.2% of cases	-The prevalence of AFS was 25.1% -72 pregnant women were at high risk for preterm birth
		AFS present—49 patients		
		Patients without AFS—146		
Fuchs et al. (2015) [40]	Case control retrospective study	126 patients	AFS group-48 had a shorter cervix	-Women with AFS delivered earlier than those from control group (p ¼ 0.03) -Smaller weight of neonates at birth (p ¼ 0.007). -A short cervix significantly associated with preterm delivery
		63 patients with AFS—study group		
		63 patients without AFS— control group		
Adanir et al. (2018) [41]	Prospective study	99 pregnant women at high risk for preterm delivery	Sensitivity of presence of AFS or CL ≤ 25 mm was 56%	-The rates of spontaneous PTB in patients with AFS at <37 weeks of gestation were 66.7% -Higher rates of neonatal morbidities in patients with AFS: 50%
		18 patients with AFS		
		74 patients without AFS		
Yasuda et al. (2020) [42]	Retrospective study	54 patients		-Delivery occurred at 28.3 ± 4.5 weeks and 31.7 ± 4.3 weeks in AFS positive patients and AFS-negative patients, respectively -Patients with AFS: 81.8%-chorioamnionitis -Patients without AFS: 20.9% chorioamnionitis -AFS did not significantly affect neonatal complications
Tsunoda et al. (2020) [43]	Retrospective cohort study	110 women (14–30 weeks pregnant and cervical length < 25 mm)	-CL-significantly shorter in the group with AFS than the group without AFS	-Increased risk of PTB in the with AFS -AFS was an independent risk factor for PTB -A significantly increased risk of PTB < 34 weeks in women with CL < 20 mm and <15 mm less than 20 and less than 15 mm
		29 women with AFS		
		81 women without AFS		
Pahlavan et al. (2022) [37]	Nested case control Study	110 pregnant women who undergone IVF procedures	<30 mm–control grup: 10.4% and in study group: 28.6%	-The prevalence of PTB in case and control group were 23.8% and 10.4% -Cerclage-in 7.5% of women from control group and 33.3% in study group
		63 patients—study group—AFS present		
		67 patients—control group—normal amniotic fluid		
Suff et al. (2023) [20]	Retrospective cohort study	147 patients with short cervical length	Women with AFS-more likely to have a short CL, 19 vs. 14 mm	-Women with AFS and short CL ≥ increased risk of mid-trimester loss and delivery <24 weeks of gestation (relative risk, 3.4; 95% confidence interval, 1.2–10.3) -Patients with AFS have increased cervicovaginal interleukin 8 concentrations and fetal fibronectin levels ≥ AFS-an indicator of an inflammatory response -Neonatal outcomes were similar between the two groups
		54 patients with AFS		
		93 patients without AFS		

**Table 2:** Studies about antibiotic therapy. Legend of Table 2: AFS = amniotic fluid sludge; US ultrasound; mg = miligrams; g = grams; OA = oral administration; IV = intravenous; VA = vaginal administration; PTB = preterm birth; vs. = versus; PROM = premature rupture of membranes; CL = cervical length.

Table 2. Reference (Author and Year)	Type of Study	Population and Study Group Assignment	Antibiotic Scheme	Outcomes
Cuff et al. (2020) [7]	Retrospective cohort study	97 Patients Included		-Did not reduce the risk of PTB -Did not improve obstetric, neonatal, or pathologic outcomes -PROM-in 33% of women who received antibiotics -PROM in 32.6% of women with no treatment -Cases without sonographic resolution were found to have a shorter CL than those with resolution-39% patients had resolution (12 had been treated and 18 had not) -61% had persistent sonographic evidence of “sludge” (23 had been treated and 24 had not)
		51 (53%) received oral antibiotics	46 patients received Azithromycin (500 mg orally on day 1, and 250 mg on days 2–5) 5 patients received Moxifloxacin (400 mg orally daily for 5 days)	
		46 (47%) were untreated.		
Hatanaka et al. (2019) [47]	Controlled observational study	86 pregnant women with singleton pregnancies and AFS present at the ultrasound	Women at low risk: Clindamycin 300 mg OA every 6 h + Cephalexin 500 mg OA every 6 h for 7 days Women at high risk: Clindamycin 600 mg IV every 8 h + Cefazolin 1 g IV every 8 h for 5 days + 5 days oral treatment	Antibiotic therapy reduced the incidence of spontaneous PTB at <34 weeks (13.2% vs. 38.5%, *p* = 0.047)
		64 pregnant women with AFS present-> divided into two subgroups: women at high risk and women at low risk		
		22 pregnant women without AFS		
Pustotina et al. (2020) [49]	Prospective Study	29 patients with AFS	All groups—IV/OA antibiotics + -vaginal antibiotic + oral probiotics for all patients (Clindamycin VA-all patients, 18 patients-butoconazole VA, 16 patients: Cefoperazone + sulbactam IV 8 patients:amoxicillin + clavulonate OA Group IIa: +vaginal progesterone Group IIb: +Indomethacin	-Neonatal complications and preterm birth were prevented in all study groups -Antibiotic therapy eliminated sonographic presence of the “sludge”
		8 women with CL > 25 mm—Group I		
		7 women with asymptomatic short cervix—Group IIa		
		14 women with short cervix and symptoms—Group IIb		
Jin et al. (2021) [46]	Retrospective cohort study	58 women at 15–23 weeks of pregnancy (uterine contractions + intact membranes + AFS present at US)	Ceftriaxone Clarithromycin Metronidazole	-Group A—a lower rate of preterm birth and neonatal morbidity -Duration of administration of antibiotics was significantly longer in group A than in group B -51.7% showed a disappearance of AFS with antibiotic treatment
		Group A (30)—disappearance of sludge		
		Group B (28)—persistent sludge		
Giles et al. (2023) [45]	Retrospective cohort study	374 women with/without AFS + cervical length of 15 mm or less at 13–24 weeks of gestation	Azithromycin	-Risk of PTB overall was higher in the treatment group -No differences found for neonatal morbidity
		129 patients received treatment		
		245 patients did not received treatment		

## Abbreviations

AFS (amniotic fluid sludge); IL (interleukin).
